# QTL Analysis of Five Silique-Related Traits in *Brassica napus* L. Across Multiple Environments

**DOI:** 10.3389/fpls.2021.766271

**Published:** 2021-11-23

**Authors:** Xiaozhen Zhao, Kunjiang Yu, Chengke Pang, Xu Wu, Rui Shi, Chengming Sun, Wei Zhang, Feng Chen, Jiefu Zhang, Xiaodong Wang

**Affiliations:** ^1^Institute of Industrial Crops, Jiangsu Academy of Agricultural Sciences, Key Laboratory of Cotton and Rapeseed, Ministry of Agriculture and Rural Affairs, Nanjing, China; ^2^State Key Laboratory of Crop Genetics and Germplasm Enhancement, Nanjing Agricultural University, Nanjing, China; ^3^College of Agriculture, Guizhou University, Guiyang, China

**Keywords:** *Brassica napus*, recombinant inbred line, silique-related traits, QTL mapping, candidate genes

## Abstract

As an important physiological and reproductive organ, the silique is a determining factor of seed yield and a breeding target trait in rapeseed (*Brassica napus* L.). Genetic studies of silique-related traits are helpful for rapeseed marker-assisted high-yield breeding. In this study, a recombinant inbred population containing 189 lines was used to perform a quantitative trait loci (QTLs) analysis for five silique-related traits in seven different environments. As a result, 120 consensus QTLs related to five silique-related traits were identified, including 23 for silique length, 25 for silique breadth, 29 for silique thickness, 22 for seed number per silique and 21 for silique volume, which covered all the chromosomes, except C5. Among them, 13 consensus QTLs, one, five, two, four and one for silique length, silique breadth, silique thickness, seed number per silique and silique volume, respectively, were repeatedly detected in multiple environments and explained 4.38–13.0% of the phenotypic variation. On the basis of the functional annotations of *Arabidopsis* homologous genes and previously reported silique-related genes, 12 potential candidate genes underlying these 13 QTLs were screened and found to be stable in multiple environments by analyzing the re-sequencing results of the two parental lines. These findings provide new insights into the gene networks affecting silique-related traits at the QTL level in rapeseed.

## Introduction

Rapeseed (*Brassica napus*, AACC, 2n = 38), a major oil crops worldwide, evolved from the double diploidization of *Brassica rapa* (AA, 2n = 20) and *Brassica oleracea* (CC, 2n = 18) through interspecific hybridization (Murphy, 1999). *B. napus* is an oil crop with the highest oil production efficiency and is currently an important source of edible vegetable oil in China (Wang, 2010). Because of the high protein content of rapeseed meal, rapeseed is also a high-quality source of animal feed. Additionally, rape straw undergoes an efficient biomass digestion and can be used as a raw material for bio-energy production (Wang X. et al., 2016).

The global population, and thus, food demand, continues to grow; however, the amount of cultivated land is decreasing owing to a variety of factors, such as human activities and climate change. Therefore, increasing the production of rapeseed is currently an urgent requirement and major goal of global rapeseed producers. Yield is an extremely complex trait, and the product of a series of developmental and physiological processes (Van Camp, 2005). Rapeseed yield is determined by three constituent factors: seed weight, silique number and seed number per silique (SPS) (Clarke and Simpson, 1978). The silique, as an important photosynthetic organ of rapeseed, is closely related to the final grain yield (Allen et al., 1971). Silique-related traits, such as silique length (SL), silique breadth (SB), silique thickness (ST) and silique volume (SV), affect the appearance and morphology of silique, and ultimately affect the production and yield of photosynthetic substances (Ferrándiz et al., 1999). Thus, siliques and their related traits are considered the major contributing factors for increasing rapeseed yield. Understanding their genetic bases is of great significance for breeding high-yield rapeseed.

The silique-related traits of rapeseed are all complex traits controlled by quantitative trait loci (QTLs), which are also easily affected by the environment. QTL mapping has been successfully applied to the genetic analyses of the quantitative traits of a variety of crops. In rapeseed, a large number of QTLs, such as those affecting plant height (Wang Y. et al., 2016; Dong et al., 2021), branch angle (Wang H. et al., 2016; Wang et al., 2019) and flowering time (Yu et al., 2019; Xu et al., 2021), have been genetically mapped. In siliques, SL and SPS are the most important traits. At present, more than 100 QTLs for SL, along with more than 200 QTLs for SPS, have been detected in different mapping populations of *B*. *napus*, and QTLs controlling SL and SPS are distributed on all 19 chromosomes, explaining 1.9–65.6% and 0.78–57.8% of the phenotypic variance (PV), respectively (Chen et al., 2007, 2011; Radoev et al., 2008; Shi et al., 2009; Zhang et al., 2011; Yang et al., 2012, 2016, 2017; Cai et al., 2014, 2016; Qi et al., 2014; Fu et al., 2015; Wang X. et al., 2016; Luo et al., 2017; Zhu et al., 2020). Recently, Li et al. (2015) successfully isolated *qSS.C9*, the main locus controlling SPS in *B. napus* that had been discovered by Zhang et al. (2012). *qSS.C9* encodes the predicted protein BnaC9.SMG7b. In *Arabidopsis*, SMG7 controls female fertility and then SPS by regulating the progression from anaphase to telophase in the second meiotic division (Riehs et al., 2008). As in *Arabidopsis*, BnaC9.SMG7b determines the formation of functional female gametophytes (FGs) by influencing the progression through meiotic anaphase II. Plants with *BnaC9.SMG7b* deletions exhibit reduced SPS and SL values, which is caused by the reduced ovule number and shorter siliques because of defects in the development of functional FGs. In addition, two major QTL for SL have been successfully cloned on the A9 chromosome of *B. napus* (Liu et al., 2015; Shi et al., 2019). The target genes of these two QTLs were identified as *BnaA9.ARF18* and *BnaA9.CYP78A9* through a fine-mapping analysis. *BnaA9.ARF18* is a homolog of the *Arabidopsis* auxin response factor 18 (*ARF18*) that is expressed differentially in various tissues, including root, leaf, stem, bud and ovule, but mainly in the silique wall. A transcription analysis has shown that *ARF18* regulates cell elongation in the silique wall and then SL by acting through the auxin-response pathway (Liu et al., 2015). *BnaA9.CYP78A9* is an ortholog of *Arabidopsis CYP78A9*, which regulates reproductive development and floral organ size (Sotelo-Silveira et al., 2013). *BnaA9.CYP78A9* affects SL in rapeseed by promoting cell elongation in silique valves during silique growth and development (Shi et al., 2019). Although a few silique genes, such as *BnaC9.SMG7b*, *BnaA9.ARF18* and *BnaA9.CYP78A9*, have been analyzed in *B. napus*, the genetic mechanisms behind silique-related traits, especially those other than SPS and SL, are far from understood.

The formation of the SPS is closely related to other silique characteristics (SL, SB, ST, and SV), and SPS is significantly positively correlated with SL (Zhang et al., 2011; Wang X. et al., 2016; Yang et al., 2017). As an important silique-related morphological trait, SL also plays a vital role in seed yield, and it has long been used as an indirect selective indicator in breeding for improved seed yields in *B. napus*. To date, there are extremely limited QTL mapping studies of SB, ST, and SV in *B. napus* (Wang X. et al., 2016), and more research is needed to determine the relationships among them and their effects on the yield, which is very important for increasing the efficiency levels of breeding programs.

In the present study, a recombinant inbred line (RIL) population containing 189 lines was used to investigate the QTLs for five silique-related traits in seven environments. The objectives were to identify (i) QTLs associated with SL, SPS, SB, ST, and SV across multiple environments and (ii) candidate genes underlying the QTLs that could be stably detected based on the re-sequencing information of two parental lines. This study will lay a good foundation for studying the molecular mechanisms of silique-related traits and increasing yield through rapeseed breeding.

## Materials and Methods

### Plant Materials

An RIL population containing 189 lines was constructed by a cross between “APL01” (female parent) and “Holly” (male parent), and it was named the AH population (Wang et al., 2015). The two parental lines showed diversity in silique-related traits. The AH population was previously used for high-density SNP genetic map construction (Wang et al., 2015). The map contains 2,755 SNP-bins, including 11,458 SNP markers and 57 simple sequence repeats, spanning a genetic distance of 2,027.53 cM, with an average distance of 0.72 cM between markers. This population was previously used for QTL mapping and the analysis of apetalous characteristics (Wang et al., 2015), seed fatty acid composition (Chen et al., 2018) and seed-related traits (Sun et al., 2018). In this study, it was used for the QTL mapping of five silique-related traits.

### Field Experiment and Trait Measurement

The AH population, and the parental lines, were grown in four locations in China in 2014–2016. Yangling, Shaanxi Province was planted in September 2015, harvested in May 2016 and recorded as 15YL; Sunan, Gansu Province, was planted in April and harvested in September 2016 and recorded as 16GS; Dali, Shaanxi Province was planted in September 2014 and 2016, harvested in May 2015 and 2017, and recorded as 14DL and 16DL, respectively; and Nanjing, Jiangsu Province was planted in September 2014, 2015 and 2016, harvested in the May of the following years and recorded as 14NJ, 15NJ, and 16NJ, respectively. The field planting of the parents and AH population followed a randomized complete block design with two replications. Each repetition contained two rows with an average spacing of 40 cm between rows and 20 cm between individual plants. The planting, management and harvesting of field materials followed local field-breeding practices.

At the mature stage, three plants growing uniformly from each line were selected, and three well-developed siliques of the first branch adjacent to the main inflorescence from each plant were collected to analyze the silique-related traits (Wang X. et al., 2016). Here, five silique-related traits were investigated: (1) SL, the average length of nine sampled siliques; (2) SB, the widest horizontal length of the space occupied by the silique; (3) ST, the thickness at the same position as SB; (4) SV, the volume measured using the drainage method, which takes the difference between the volume reading of the water after silique immersion and the volume reading of the water prior to the immersion; and (5) SPS, the average seed number of nine sampled siliques. A Vernier caliper was used to measure SL, SB and ST, and a volumetric cylinder was used to measure SV.

### Statistical Analysis

SPSS 22.0 software (SPSS Inc., Chicago, IL, United SA) was used for descriptive statistical and correlation analyses of each trait in each environment, and the mean value of the seven environments for each trait was used to carry out correlation analyses. The coefficient of variation was calculated as σ/μ, where σ represents the standard deviation and μ represents the average. The broad-sense heritability (*h*^2^) was obtained using the R package lme4 (Merk et al., 2012). The computational formula for broad-sense heritability was as follows: *h*^2^ = σ^2^_*G*_/(σ^2^_*G*_ + σ^2^_*GE*_/*e* + σ^2^_*e*_/*re*), where σ^2^_*G*_, σ^2^_*GE*_, and σ^2^_*e*_, respectively, represent genotypic variance, the interaction variance of genotype-by-environment and error variance, n represents the number of environments and r represents the number of replications (Sun et al., 2016).

### QTL Mapping and Integration

The composite interval mapping procedure of the Windows QTL Cartographer 2.5 was used to detect the QTLs associated with the five silique-related traits (Wang et al., 2012). The window size was set to 10 cM, a walking speed of 2 cM was selected, and five markers were set as background cofactors. The permutation analysis with 1,000 repetitions (*P* = 0.05) was performed, and a LOD threshold (2.5–2.8) was used to determine the existence of a QTL and named as “identified QTL.” The QTL intervals were established by 2-LOD as approximately 95% QTL confidence intervals (CIs), which is automatically generated by Windows QTL Cartographer 2.5. Identified QTLs for each trait have undergone two rounds of meta-analysis using BioMercator 2.1 software, which can be used to decide the best fitting QTL in accordance with the Akaike criterion (Arcade et al., 2004). The QTL meta-analysis was performed following the description of Wang X. et al. (2016). In the first round, identified QTLs for specific traits with overlapping CIs detected in two or more environments were integrated and named as “consensus QTLs.” In the second round, consensus QTLs for different traits located in the same chromosomal region were integrated and named as “unique QTLs” (Chen et al., 2018). The designation of the QTL refers to the method of McCouch et al. (1997) with modifications. The name of identified QTL starts with its abbreviation “*iq*,” followed by the environment/trait abbreviation and linkage group (A1–10 and C1–9). When there are multiple QTLs within the same linkage group, the identified QTLs are numbered in accordance with their physical locations. Similarly, the names of consensus QTLs and unique QTLs start with their abbreviations “*cq*” and “*uq*,” respectively.

### Prediction of Underlying Candidate Genes for SL, SB, ST, SPS, and SV

The Illumina HiSeq 2500 platform (Illumina, Inc., San Diego, CA, United States) was used to re-sequence the parental APL01 and Holly genomes with a sequencing depth of 30 × coverage in our previous study (Yu et al., 2021). A total of 74.89 G bp clean data were obtained, with Q30 reaching an average of 92.0%. For candidate gene prediction the following steps were taken: First, the SNP/Indel loci with differences and homozygous between the parents were detected. The clean reads of APL01 and Holly were aligned against *B*. *napus* reference genome “Darmor-*bzh*” (Chalhoub et al., 2014) using BWA software (Li and Durbin, 2009). The SAMtools command “mpileup” was used to genotypes calling (Li et al., 2009). The locus were screened and retained with criteria: (1) Depth ≥ 6; (2) Quality ≥ 20; (3) Allele mutation ratio of homozygous loci <0.1 or >0.9. After filtration, the genotypes of the two parents were combined by the Bcftools command “merge” (Danecek and McCarthy, 2017), and the SNP/Indel loci with differences and homozygous between the parents were retained; Second, the probe sequences of SNPs on the genetic map were used to map markers on both sides of the QTL interval to the physical position of the reference genome “Darmor-*bzh*” using BLAST (E value ≤ 1e-10) (Chalhoub et al., 2014), and the variation loci within the QTL interval were found using the results of the first step; and Third, on the basis of the functional annotations of *Arabidopsis* homologous genes and previously reported silique-related genes, candidate genes for silique-related traits of rapeseed were screened from the mutant loci of these QTLs.

## Results

### Phenotypic Analysis of Five Silique-Related Traits

The phenotypic performance and *h*^2^ estimates for the five silique-related traits in the two parental lines and AH population are presented in Table 1. The five silique-related traits of the two parents, “APL01” and “Holly,” differed significantly in most of the investigated environments, and only two environments, 16DL and 16NJ, showed no significant differences in ST. Compared with “Holly,” “APL01” had significantly greater SB, ST, SV, and SPS values, whereas “Holly” had a significantly greater average SL than “APL01.” The *h*^2^ values for SL, SB, ST, SV, and SPS were 81.10, 82.45, 76.66, 73.10, and 73.61% in the AH population, respectively (Table 1), suggesting that the QTLs controlling these traits have large effects on breeding rapeseed to increase yield.

**TABLE 1:** Statistical analysis of five silique-related traits for two parents and AH populations in 7 environments.

		Parental line		Pt-test	RILs					
Trait	Treatment	APL01 (mean ± SD)	Holly (mean ± SD)		Mean ± SD	Range	CV	Skewness	Kurtosis	*h*^2^ (%)
SL (cm)	14DL	6.02 ± 0.58	7.14 ± 0.48	6.77E-04**	6.23 ± 0.76	4.0-9.0	0.12	0.08	–0.11	81.10
	14NJ	6.33 ± 0.27	6.69 ± 0.22	1.06E-02*	6.20 ± 0.75	3.5-8.6	0.12	0.02	0.58	
	15NJ	5.92 ± 0.38	6.47 ± 0.36	9.69E-03*	6.34 ± 0.76	3.9-9.3	0.12	0.05	–0.04	
	15YL	5.30 ± 0.65	5.86 ± 0.31	4.43E-02*	6.15 ± 0.78	4.0-10.7	0.13	0.72	2.72	
	16DL	5.41 ± 0.42	6.04 ± 0.32	4.00E-03**	6.21 ± 0.81	3.7-9.1	0.13	–0.02	0.20	
	16GS	6.12 ± 0.56	7.01 ± 0.52	5.00E-03**	6.38 ± 0.75	4.2-9.0	0.12	0.18	0.02	
	16NJ	5.60 ± 0.19	6.62 ± 0.55	1.41E-04**	6.19 ± 0.79	1.5-10.8	0.13	0.37	2.42	
SB (mm)	14DL	5.56 ± 0.08	4.34 ± 0.21	1.58E-03**	4.58 ± 0.43	3.2-6.6	0.09	0.39	1.23	82.45
	14NJ	5.55 ± 0.13	4.51 ± 0.40	2.46E-02*	4.84 ± 0.47	3.2-6.0	0.10	–0.05	0.40	
	15NJ	5.63 ± 0.20	4.44 ± 0.24	5.65E-03**	5.01 ± 0.50	3.5-7.0	0.10	0.20	–0.01	
	15YL	5.57 ± 0.13	4.42 ± 0.23	3.44E-03**	4.93 ± 0.46	3.7-6.6	0.09	0.36	0.24	
	16DL	5.36 ± 0.31	4.15 ± 0.21	9.96E-03**	4.69 ± 0.45	3.6-6.0	0.10	0.12	–0.15	
	16GS	5.56 ± 0.19	4.49 ± 0.21	5.77E-03**	4.93 ± 0.47	3.6-7.2	0.10	0.74	2.09	
	16NJ	5.08 ± 0.17	4.04 ± 0.45	3.00E-03**	4.91 ± 0.57	3.3-7.1	0.12	0.39	0.36	
ST (mm)	14DL	3.81 ± 0.13	3.36 ± 0.11	2.19E-02*	3.34 ± 0.28	2.6-4.2	0.08	0.29	0.25	76.66
	14NJ	3.75 ± 0.18	3.33 ± 0.02	2.68E-02*	3.42 ± 0.33	2.5-4.5	0.10	0.28	0.15	
	15NJ	3.84 ± 0.06	3.50 ± 0.10	1.38E-02*	3.51 ± 0.33	2.7-5.3	0.09	0.46	0.84	
	15YL	3.67 ± 0.03	3.46 ± 0.09	3.14E-02*	3.48 ± 0.30	2.4-4.7	0.09	0.22	0.52	
	16DL	3.70 ± 0.09	3.37 ± 0.14	5.43E-02	3.48 ± 0.29	2.7-4.6	0.08	0.40	0.73	
	16GS	3.70 ± 0.07	3.49 ± 0.06	3.24E-02*	3.52 ± 0.31	2.6-4.8	0.09	0.17	0.52	
	16NJ	3.72 ± 0.11	3.57 ± 0.14	3.27E-01	3.41 ± 0.42	2.2-5.8	0.12	0.86	1.99	
SV (ml)	14DL	0.88 ± 0.10	0.56 ± 0.01	1.29E-02*	0.68 ± 0.14	0.3-1.2	0.21	0.46	0.52	73.10
	14NJ	0.92 ± 0.08	0.61 ± 0.07	7.30E-05**	0.70 ± 0.17	0.3-1.2	0.24	0.34	–0.06	
	15NJ	0.98 ± 0.03	0.66 ± 0.03	6.08E-04**	0.80 ± 0.15	0.4-1.5	0.19	0.80	1.84	
	15YL	0.98 ± 0.06	0.60 ± 0.03	9.32E-04**	0.79 ± 0.16	0.5-1.5	0.20	1.18	3.67	
	16DL	0.91 ± 0.03	0.41 ± 0.07	6.78E-04**	0.66 ± 0.14	0.3-1.3	0.20	0.60	0.86	
	16GS	0.99 ± 0.08	0.60 ± 0.00	2.41E-03**	0.86 ± 0.14	0.5-1.7	0.17	1.02	3.77	
	16NJ	0.91 ± 0.07	0.50 ± 0.10	5.80E-5**	0.64 ± 0.18	0.2-1.3	0.29	0.49	0.45	
SPS	14DL	25.0 ± 2.8	19.7 ± 2.1	4.91E-04**	22.5 ± 4.23	2-37	0.19	–0.17	0.38	73.61
	14NJ	27.9 ± 2.8	22.4 ± 2.1	4.64E-04**	21.9 ± 4.25	2-36	0.19	–0.27	0.69	
	15NJ	28.0 ± 4.8	23.6 ± 1.8	2.65E-02*	23.1 ± 5.00	5-38	0.22	–0.25	0.00	
	15YL	22.7 ± 3.0	18.3 ± 2.7	7.75E-03**	22.4 ± 4.57	9-42	0.20	0.04	0.00	
	16DL	21.4 ± 1.9	17.9 ± 1.5	9.02E-04**	21.9 ± 4.67	2-38	0.21	–0.01	0.25	
	16GS	30.6 ± 3.5	26.8 ± 2.3	2.20E-02*	24.4 ± 4.05	2-40	0.17	–0.13	0.66	
	16NJ	25.9 ± 2.64	15.7 ± 2.21	3.0E-7**	22.3 ± 4.28	2-35	0.19	–0.09	–0.13	

*SL, SB, ST, SPS and SV indicate the traits silique length, silique breadth, silique thickness, seed number per silique and silique volume, respectively.*

*DL, Dali; NJ, Nanjing; YL, Yangling; GS, Gansu; 14, 15, and 16 indicate the years 2014, 2015 and 2016, respectively.*

**Significant at the 0.01 probability level.

*Significant at the 0.05 probability level.

*CV, Coefficient of variation.*

**h*^2^, Broad-sense heritability.*

**FIGURE 1:**
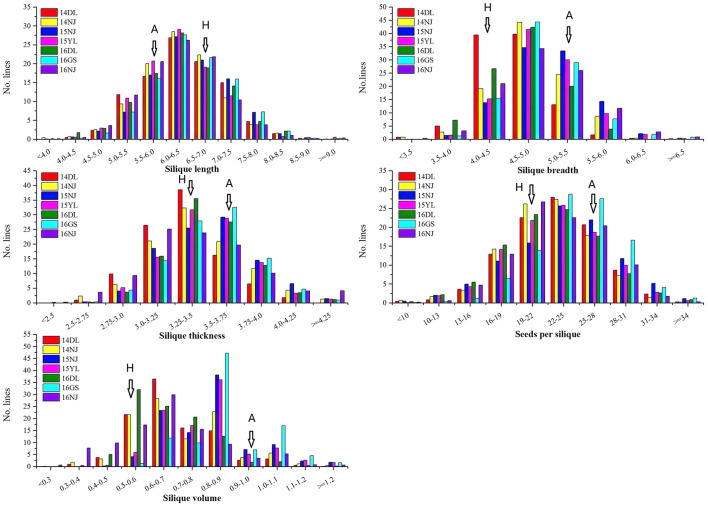
Phenotype frequency distributions of five silique-related traits in the AH population. 14DL, 14NJ, 15NJ, 15YL, 16DL, 16GS, and 16NJ are the codes for the following different years and environments: 2014 Dali, 2014 Nanjing, 2015 Nanjing, 2015 Yangling, 2016 Dali, 2016 Gansu, and 2016 Nanjing, respectively. A represents the female parent “APL01” and H represents the male parent “Holly” of the AH population.

A wide range of variation, as well as transgressive segregation, were observed for the five silique-related traits, suggesting that alleles with positive effects were distributed in both parents (Table 1 and Figure 1). In addition, the distributions of the five traits were continuous, and most skewness and kurtosis values for the distributions of these traits were <1.0 (Table 1 and Figure 1), which are characteristic of the normal distribution model, indicating that the AH population is suitable for QTL mapping.

### Correlation Analysis Among Five Silique-Related Traits

The correlation coefficients among the five silique-related traits were calculated (Table 2), and most were extremely significantly correlated with each other. For instance, highly significant positive correlations were observed between SV and the other four silique-related traits, SB was significantly positively correlated with ST, SPS and SV, and SPS showed significant positive correlations with the other silique-related traits, except for ST. Studying the associations among SL, SB, ST, SPS, and SV is of great significance to understand silique morphogenesis and increase rapeseed yield.

**TABLE 2:** Phenotypic correlations among five silique-related traits in the AH population.

Trait	SL	SB	ST	SPS	SV
SL	1				
SB	0.040	1			
ST	–0.026	0.489**	1		
SPS	0.571**	0.268**	–0.027	1	
SV	0.554**	0.718**	0.582**	0.498**	1

*SL, SB, ST, SPS, and SV indicate the traits silique length, silique breadth, silique thickness, seed number per silique, and silique volume, respectively.*

**Significant at the 0.01 probability level.

### QTL Mapping of Five Silique-Related Traits

**FIGURE 2:**
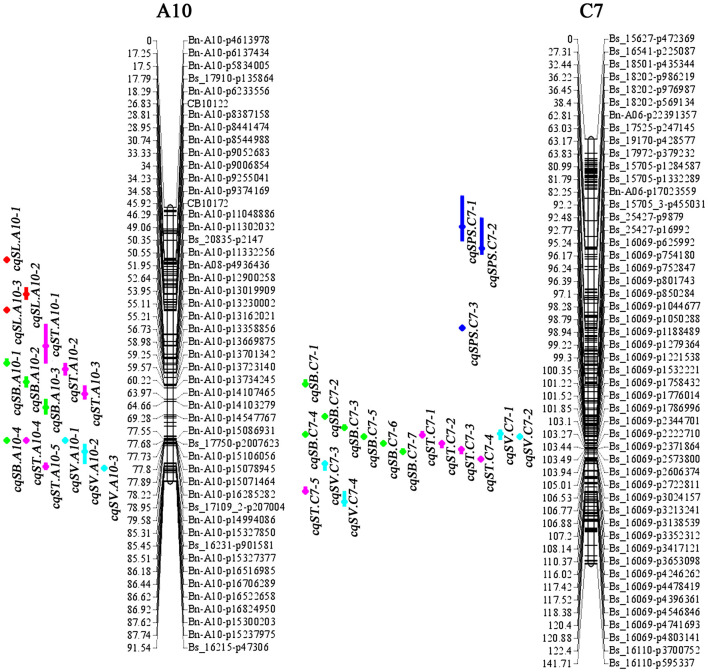
Consensus QTL locations of silique-related traits on chromosomes A10 and C7 detected in seven different environments. Only SNPs in the confidence interval of each QTL, and the terminal two SNPs of each linkage group, are marked. The numbers listed on the left of the linkage group are distances in cM, and the locus names are on the right. Consensus QTLs for different traits are represented by different colored vertical bars: red, SL; green, SB; pink, ST; blue, SPS; turquoise, SV).

Genome-wide QTL analyses were performed separately using the phenotypic data of SL, SB, ST, SPS, and SV grown in seven environments, and a total of 137 identified QTLs distributed across 18 chromosomes were obtained, which explained 4.23–15.31% of the PV (Supplementary Additional File 1). Among the QTLs, 24 were identified for SL, 33 for SB, 31 for ST, 27 for SPS, and 22 for SV (Supplementary Additional File 1). For each trait, QTLs with overlapping CIs detected in different environments were integrated into one QTL through a meta-analysis. As a result, 137 identified QTLs were integrated into 120 consensus QTLs (Figure 2, Supplementary Additional Files 1, 2). Among them, 107, 10, 2 and 1 consensus QTL were detected in 1, 2, 3, and 4 environments, respectively. The consensus QTLs repeatedly detected in multiple environments are listed in Table 3.

**TABLE 3:** Consensus QTL obtained for the five silique-related traits detected in multiple environments.

Trait	QTL	Chr.	Position (cM)	CI. (cM)	LOD	Add	R2%	Env.
SL	*cqSL.A10-3*	A10	34.21	33.66–34.76	2.73–4.07	0.15–0.17	5.25–7.22	14DL/15YL
SB	*cqSB.A10-2*	A10	58.22	56.5–59.9	3.19–5.2	−0.12	6.08–9.04	14DL/16DL/16NJ
	*cqSB.C1*	C1	11.6	10.7–12.5	2.76–3.42	0.10–0.11	4.61–6.22	15YL/16GS
	*cqSB.C6-1*	C6	51.25	50.2–52.4	2.5–6.98	0.11–0.15	4.38–13.0	14DL/14NJ/15NJ/16DL
	*cqSB.C7-2*	C7	92.57	92.16–92.97	4.97–5.79	0.14–0.2	9.22–11.7	15YL/16GS
	*cqSB.C7-4*	C7	98.52	98.1–99.0	3.91–5.26	0.11–0.14	6.73−9.63	16DL/16GS
ST	*cqST.A5-2*	A5	22.15	21.2−23.11	2.69−4.03	−0.06–−0.07	5.04–7.52	16DL/16GS
	*cqST.A5-3*	A5	29.04	27.69–30.39	3–3.14	−0.07	5.91–6.16	14DL/14NJ
SPS	*cqSPS.A7-3*	A7	74.09	72.21–75.98	3.61–6.29	0.85–1.26	6.92–11.2	14DL/16DL
	*cqSPS.C3-2*	C3	33.97	28.09–39.84	2.66–3.17	0.8–0.97	5.49–5.75	15NJ/16GS
	*cqSPS.C3-3*	C3	41.27	41.08–41.46	3.16–4.47	0.9–0.96	5.76–8.25	15NJ/15YL/16NJ
	*cqSPS.C6-2*	C6	52.02	50.03–54.01	2.97–3.14	0.78–0.89	5.93–6.13	15YL/16NJ
SV	*cqSV.A10-1*	A10	77.7	77.61–77.8	3.09–4.44	−0.03–−0.04	6.5–8.4	14DL/15NJ

*SL, SB, ST, SPS, and SV indicate the traits silique length, silique breadth, silique thickness, seed number per silique, and silique volume, respectively.*

*Chr., Chromosome.*

*CI., Confidence interval (cM).*

*Add, Additive effect.*

*Env., The experiment in which the consensus QTLs were detected.*

For the 24 identified QTLs of SL, which were located on chromosomes A3, A5, A6, A7, A9, A10, C2, C3, C6, and C9, each individual QTL explained 4.8–9.2% of the PV. Two identified QTLs, *iq14DL.A10-2* and *iq15YL.A10*, with overlapping CIs were integrated into the consensus QTL *cqSL.A10-3* (Table 3). Among the 23 consensus QTLs after integration, all the QTLs showed positive additive effects, except *cqSL.A5-3*, suggesting that the female parent “APL01” contributed favorable alleles.

For the 33 identified QTLs of SB, each QTL accounted for 4.23–15.3% of the PV, and these QTLs were mapped onto 12 chromosomes. Finally, the 33 identified QTLs were integrated into 25 consensus QTLs, including 3, 1 and 1 QTLs stably expressed in 2, 3 and 4 environments, respectively (Table 3). The stable QTL *cqSB.C6-1* showed a larger effect in the DL than in the NJ environment, with PVs of 12.96 and 9.53% in 14DL and 16DL, respectively but PVs of 4.38 and 5.18% in 14NJ and 15NJ, respectively. The QTL *cqSB.C6-1* was repeatedly detected in four environments and may be used in marker-assisted selection. Four of the five consensus QTLs (*cqSB.C1*, *cqSB.C7-2*, *cqSB.C7-4*, and *cqSB.C6-1*) repeatedly detected in different environments had positive additive effects, indicating that the alleles responsible for increasing SB were inherited from female parent “APL01.”

For the 31 identified QTLs of ST, each QTL explained 4.5–9.5% of the PV and were located on ten chromosomes. The 31 QTLs were integrated into 29 consensus QTLs, including two QTLs that were repeatedly detected in two environments. QTL *cqST.A5-2* was integrated from *iq16DL.A5* and *iq16GS.A5*, whereas *cqST.A5-3* was integrated from *iq14DL.A5-1* and *iq14NJ.A5-2* (Table 3). These two consensus QTLs had negative additive effects, indicating that the favorable alleles were derived from the male parent “Holly.”

For SPS, 27 identified QTLs were obtained, explaining 4.5–13.8% of the PV. These QTLs were located on nine chromosomes. As a result of meta-QTL analyses, 22 consensus QTLs were obtained, including 18, 3 (*cqSPS.A7-3*, *cqSPS.C3-2*, and *cqSPS.C6-2*) and 1 (*cqSPS.C3-3*) QTL that were expressed in 1, 2, and 3 environments, respectively (Table 3). Among the 22 consensus QTLs, only four had negative additive effects and exhibited minor effects, whereas the other 18 QTLs had positive additive effects, indicating that the female parent “APL01” contributed favorable alleles.

For SV, 22 identified QTLs were detected, with each QTL explaining 4.62–11.9% of the PV. These QTLs were distributed across 13 chromosomes. There are two identified QTLs, *iq14DL.A10-1* and *iq15NJ.A10*, with overlapping CIs that were integrated into one consensus QTL, *cqSV.A10-1* (Table 3), and the other QTLs were only specifically expressed in a single environment (Supplementary Additional File 1). Of the 21 consensus QTLs, 15 had positive additive effects, including the two QTLs, *cqSV.C7-2* and *cqSV.C7-3*, which explained 10.3 and 11.9%, respectively, of the PV in 16GS, indicating that the female parent “APL01” contributed favorable alleles.

### Pleiotropic Unique QTLs for the Five Silique-Related Traits

As shown in Table 2, most of the five silique-related traits showed highly significant correlations. The genetic correlations may be caused by pleiotropism, in which, a single QTL affects the phenotypic variation of multiple traits. Consensus QTLs for the five silique-related traits with overlapping CIs were integrated into unique QTLs using a meta-analysis. In total, 120 consensus QTLs were integrated into 89 unique QTLs (Supplementary Additional File 3), including 25 QTLs that had pleiotropic effects on two to three traits (Table 4). These pleiotropic QTLs can partly explain the significant correlations between silique-related traits. For instance, SB had a significant positive correlation with SV, with the highest positive correlation coefficient (0.718) among the five silique-related traits (Table 2). Five unique QTLs simultaneously controlled SB and SV, in which *uqC6-4*, *uqC7-7*, *uqC7-8*, and *uqC8* had positive additive effects, while *uqA10-8* had a negative effect on both SB and SV (Table 4).

**TABLE 4:** Twenty-five pleiotropic unique QTL on two to three traits of silique-related traits obtained by the second round of meta-analysis.

Unique QTLs			Consensus QTLs								
QTL	Position (cM)	CI. (cM)	QTL	Chr.	Position (cM)	CI. (cM)	LOD	Add	R2%	Env.	Trait
*uqA1-2*	44.05	41.56–46.54	*cqSPS.A1-1*	A1	42.21	41.2–47	2.83	−0.72	5.11	16NJ	SPS
			*cqSB.A1-2*	A1	49.21	46.2–55.9	3.35	0.11	5.64	16GS	SB
*uqA2-3*	96.6	93.88–99.32	*cqSV.A2-3*	A2	96.21	92.2–99	2.84	−0.04	5.8	16NJ	SV
			*cqST.A2-1*	A2	97.31	90.8–99.9	2.53	−0.05	4.63	15YL	ST
*uqA4-2*	60.9	58.95–62.84	*cqSPS.A4*	A4	57.31	56–62.4	4.02	1.07	7.29	15NJ	SPS
			*cqST.A4*	A4	63.01	62.3–67.2	2.72	0.06	5.13	16GS	ST
*uqA5-4*	22.81	21.97–23.64	*cqST.A5-2*	A5	22.15	21.2–23.11	2.69∼4.03	−0.06∼0.71	5.04∼7.52	16DL/16GS	ST
			*cqSPS.A5-1*	A5	24.91	22.1–25.5	3.02	0.71	5.71	14DL	SPS
*uqA7-3*	67.53	66.89–68.17	*cqSPS.A7-2*	A7	66.61	64.2–67.4	4.49	1.09	8.44	16DL	SPS
			*cqSL.A7-1*	A7	67.71	66.6–68	2.93	0.17	5.78	16GS	SL
*uqA7-5*	70.53	70.35–70.72	*cqSB.A7-2*	A7	70.01	69.7–70.8	5	−0.14	9.12	14DL	SB
			*cqSL.A7-2*	A7	70.61	70.4–70.8	3.11	0.16	6.03	16GS	SL
*uqA7-6*	74.81	73.21–76.41	*cqSPS.A7-3*	A7	74.09	72.21–75.98	3.61∼6.29	0.85∼1.26	6.92∼11.2	14DL/16DL	SPS
			*cqSB.A7-3*	A7	76.71	73.8–79.9	4.02	−0.13	7.79	14DL	SB
*uqA9-1*	10.03	5.95–14.11	*cqSL.A9*	A9	5.31	3.6–15	2.56	0.15	4.8	16DL	SL
			*cqSB.A9*	A9	15.01	8.1–19.8	3.77	−0.13	7.1	15NJ	SB
*uqA10-4*	52.32	51.36–53.28	*cqST.A10-1*	A10	46.31	38.9–52	3.81	−0.069	7.05	16DL	ST
			*cqSB.A10-1*	A10	52.01	50.4–52.6	5.43	−0.13	10.1	14DL	SB
			*cqST.A10-2*	A10	54.01	52–56.1	3.75	−0.07	7.07	16DL	ST
*uqA10-8*	77.7	77.61–77.78	*cqSV.A10-1*	A10	77.7	77.61–77.8	3.09∼4.44	−0.0329∼−0.04	6.5∼8.4	14DL/15NJ	SV
			*cqSB.A10-4*	A10	77.71	77.6–78.2	2.62	−0.1	4.84	15NJ	SB
			*cqST.A10-4*	A10	77.71	77.6–78.2	4.39	−0.08	8.34	15NJ	ST
*uqA10-10*	86.83	86.33–87.33	*cqST.A10-5*	A10	86.51	85.3–87.6	3.42	−0.07	6.43	15NJ	ST
			*cqSV.A10-3*	A10	86.91	86.6–87.7	4.48	−0.0336	8.62	14DL	SV
*uqC2-3*	52.45	51.08–53.83	*cqSPS.C2-2*	C2	51.01	49.9–53.8	3.65	0.93	7.14	16GS	SPS
			*cqSV.C2*	C2	53.91	53.1–57	2.56	0.03	4.98	16GS	SV
*uqC3-1*	17.2	14.41–20.0	*cqSL.C3-1*	C3	17.21	11.7–23.2	3.34	0.17	6.3	16DL	SL
			*cqSPS.C3-1*	C3	17.21	13.9–20.7	7.54	1.3	13.8	16DL	SPS
			*cqSV.C3*	C3	17.21	7.9–27.1	2.6	0.05	4.78	16DL	SV
*uqC3-4*	41.33	41.15–41.51	*cqSPS.C3-3*	C3	41.27	41.08–41.46	3.16∼4.47	0.9∼0.96	5.76∼8.25	15NJ/15YL/16NJ	SPS
			*cqSL.C3-2*	C3	42.11	41.4–42.7	2.82	0.16	5.45	15YL	SL
*uqC3-6*	50.05	48.67–51.43	*cqSPS.C3-5*	C3	50.01	48.1–51	3.31	0.79	6.2	16NJ	SPS
			*cqSB.C3*	C3	50.51	50.3–59.3	3.46	0.12	6.39	15NJ	SB
*uqC6-3*	43.21	41.3 - 45.11	*cqSPS.C6-1*	C6	43.21	41.2–46	4.53	0.91	8.31	16NJ	SPS
			*cqSV.C6-1*	C6	43.21	40.9–47.2	2.85	0.05	5.29	16DL	SV
*uqC6-4*	51.45	50.55–52.36	*cqSB.C6-1*	C6	51.25	50.14–52.37	2.5∼6.98	0.11∼0.15	4.38∼12.96	14DL/14NJ/15NJ/16DL	SB
			*cqSV.C6-2*	C6	51.61	50.9–55.9	3.15	0.06	5.89	16DL	SV
			*cqSPS.C6-2*	C6	52.02	50.03–54.01	2.97∼3.14	0.78∼0.89	5.93∼6.13	15YL/16NJ	SPS
*uqC7-7*	98.59	98.21–98.96	*cqSV.C7-1*	C7	98.31	97.1–100.3	4.15	0.07	7.77	16DL	SV
			*cqSB.C7-4*	C7	98.52	98.06–98.97	3.91∼5.26	0.11∼0.14	6.73∼9.63	16DL/16GS	SB
			*cqST.C7-1*	C7	98.81	97.6–99	4.13	0.07	7.79	16DL	ST
*uqC7-8*	99.2	99.12–99.29	*cqSB.C7-5*	C7	99.21	99.1–99.3	7.25	0.2	14.16	15YL	SB
			*cqSV.C7-2*	C7	99.21	99–99.3	5.15	0.04	10.3	16GS	SV
*uqC7-9*	101.51	101.18–101.83	*cqSB.C7-6*	C7	101.51	101.2–101.9	5.81	0.15	10.58	16GS	SB
			*cqST.C7-2*	C7	101.51	101.2–103.1	2.76	0.08	5.42	14NJ	ST
*uqC7-10*	103.51	103.3–105.0	*cqST.C7-3*	C7	103.51	103.3–105	3.11	0.07	5.85	16GS	ST
			*cqSB.C7-7*	C7	104.11	103.3–104.5	7.54	0.22	14.63	15YL	SB
*uqC7-13*	117.89	116.96–118.82	*cqST.C7-5*	C7	117.41	116–118	3.23	−0.1	6.2	14NJ	ST
			*cqSV.C7-4*	C7	120.91	117.5–122.5	3.99	0.031	7.54	14DL	SV
*uqC8*	14.19	13.71–14.67	*cqSB.C8*	C8	14.11	13.4–14.4	3.45	0.12	6.37	15NJ	SB
			*cqSV.C8*	C8	15.01	14.8–24.6	2.62	0.03	4.98	15NJ	SV
			*cqSPS.C8*	C8	15.11	14.4–17.9	3.77	1.01	6.84	15NJ	SPS
*uqC9-5*	115.47	114.65–116.28	*cqST.C9-4*	C9	115.11	113–115.6	3.54	0.065	6.56	15YL	ST
			*cqSB.C9*	C9	115.71	114.2–116.3	5.25	0.14	9.96	15YL	SB
*uqC9-6*	117.01	116.02–117.99	*cqST.C9-5*	C9	117.01	116.4–118.8	3.27	0.08	5.99	16NJ	ST
			*cqSV.C9*	C9	117.01	116.3–119.8	2.54	0.03	4.72	15YL	SV

*SL, SB, ST, SPS, and SV indicate the traits silique length, silique breadth, silique thickness, seed number per silique, and silique volume, respectively.*

*DL, Dali; NJ, Nanjing; YL, Yangling; GS, Gansu; 14, 15, and 16 indicate the years 2014, 2015 and 2016, respectively.*

*Chr., Chromosome.*

*CI., Confidence interval (cM).*

*Add, Additive effect.*

*Env., The experiment in which the consensus QTLs were detected.*

### Prediction of Candidate Genes for SL, SB, ST, SPS, and SV

To identify candidate genes related to SL, SB, ST, SPS, and SV, 13 consensus QTLs for the five traits that were stably expressed in multiple environments (Table 3) were analyzed. As a result, 2–271 genes underlying the CIs of the 13 QTLs were found (Supplementary Additional File 4). We analyzed these 2–271 genes one by one based on the functional annotations of *Arabidopsis* homologous genes and previously reported silique-related genes. Twelve genes underlying 12 consensus except for *cqSV.A10-1* were found to have known functions related to the silique-related traits.

The CI of *cqSL.A10-3* contained the putative gene *SMG7* (*BnaA10g15730D*), which have been successively cloned by Li et al. (2015). The CI of *cqSB.A10-2* contained the putative genes *LNG1* (*BnaA10g18650D*) and *CNGC18* (*BnaA10g19030D*), *lng1* dominant mutant displayed longitudinally elongated and transversely narrowed in cells of the siliques (Lee et al., 2006), the *CNGC18* point mutations resulted in shorter siliques, reduced male fertility and fewer seeds per silique (Gao Q. et al., 2016). The CI of *cqSB.C1* contained the putative gene *STY2* (*BnaC01g02360D*), the *sty2* mutation prevents siliques from elongating and showed short silique in *Arabidopsis* (Kuusk et al., 2002). The CI of *cqSB.C7-2* contained the putative gene *KIN*βγ (*BnaC07g33610D*), its mutant displayed defects in organogenesis and growth, including shorter stature and silique (Gao X. et al., 2016). The CI of *cqSB.C7-4* contained the putative gene *ABCC13* (*BnaC07g35090D*), *ABCC13* loss function in *Arabidopsis* leads to decreased silique length and seed yield (El Guizani et al., 2014). The CI of *cqST.A5-2* contained the putative gene *ARID1* (*BnaA05g05310D*), mutation in *ARID1* showed reduced seed set and short siliques (Zheng et al., 2014). The CI of *cqST.A5-3* contained the putative gene *LAC4* (*BnaA05g06610D*), the overexpression of *miR397b* in *Arabidopsis* showed increased silique length and seed yield via modulating gene *LAC4* (Wang et al., 2014). The CI of *cqSPS.A7-3* contained the putative gene *HTH* (*BnaA07g22900D*), *hth* mutant showed reduced seed set and short siliques (Krolikowski et al., 2003). The CI of *cqSPS.C3-2* contained the putative gene *AOG1* (*BnaC03g12770D*), the siliques of *aog1* mutant were much smaller than those of wild type and had a dramatically reduced seed set (Cui et al., 2015). The CI of *cqSPS.C3-3* contained the putative gene *PYL8* (*BnaC03g15210D*), the *pyl8* T-DNA mutant had an extremely low seed yield due to fewer siliques with lower length (Gonzalez-Guzman et al., 2012). In this study, no candidate genes were discovered in the CI of *cqSV.A10-1*. The CI of *cqSB.C6-1* and *cqSPS.C6-2* contained the putative gene *HMG1* (*BnaC06g26470D*), T-DNA insertion *hmg1* mutant showed shorter siliques and fewer seeds per silique in *Arabidopsis* (Suzuki et al., 2004).

## Discussion

Most agricultural production-related traits are quantitative traits controlled by multiple genes that are also sensitive to the environmental conditions (Abe et al., 2012). Therefore, QTL detection across different environments is necessary for the genetic dissection of complex traits in crops. In this study, phenotypic observations of five silique-related traits replicated in seven environments were used for the QTL analysis, and they might improve the accuracy of QTL detection.

In the present study, most of the five silique-related traits have significant positive correlations with each other (Table 2). The significant correlations can be explained by unique pleiotropic QTLs. For example, SB had significant positive correlations with SV (coefficient 0.718), whereas, five unique QTLs simultaneously effect SB and SV with the same direction of additive effects (Supplementary Additional File 3). ST showed a negative but low correlations with SL (coefficient -0.026) and SPS (coefficient −0.027) (Table 4). Accordingly, no unique QTL had effects on both ST and SL, whereas only two unique QTLs controlled ST and SPS. These were *uqA4-2*, with the same direction of additive effects, and *uqA5-4*, with the opposite direction of additive effects. In addition, SPS had significant positive correlations with SL, SB and SV, but not ST, which is consistent with previous results (Zhang et al., 2011; Wang X. et al., 2016; Yang et al., 2017).

In this study, 25, 29, and 21 consensus QTLs were obtained for SB, ST and SV, respectively. Among these QTLs, five for SB, two for ST and one for SV were repeatedly identified in two to four different environments. To our knowledge, QTLs for SB, ST, and SV in *B*. *napus* have rarely been reported (Wang X. et al., 2016). In this study, the QTLs for SB were first mapped on chromosomes A1, A10, C1, C3, C4, C8, and C9; QTLs for ST were first mapped on chromosomes A2, A4, C3, and C7; and QTLs for SV were first mapped on chromosomes A2, A4, A7, A8, A10, C2, C3, C4, C7, C8, and C9. Meanwhile, in previously published results, two QTLs related to SB located at 60.47–62.39 Mb and 68.69–72.5 Mb of C6 had overlapping CIs with the results of our study (*cqSB.C6-2*, 60.6–61.9 Mb and *cqSB.C6-3*, 70.1–83.3 Mb), two QTLs related to ST located at 61.3–69.2 Mb of A9 and 34.1–60.4 Mb of A10 had overlapping CIs with the results of our study (*cqST.A9-2*, 68.1–75 Mb and *cqST.A10-1*, 38.9–52 Mb) and one QTL related to SV located at 54.8–60.7 Mb of C6 had an overlapping CI with the results of our study (*cqSV.C6-2*, 50.9–55.9 Mb). This indicated that these QTLs were stably expressed in different genetic backgrounds and that they may be useful in molecular marker-assisted selection breeding.

In previous studies, efforts focused on QTL analyses of SPS and SL. There are more than 200 QTLs for SPS (Radoev et al., 2008; Shi et al., 2009; Chen et al., 2011; Zhang et al., 2011; Cai et al., 2014, 2016; Qi et al., 2014; Wang X. et al., 2016; Yang et al., 2016, 2017; Luo et al., 2017; Zhu et al., 2020) and more than 100 QTLs for SL located on 19 chromosomes (Chen et al., 2007; Zhang et al., 2011; Yang et al., 2012, 2017; Cai et al., 2014; Qi et al., 2014; Fu et al., 2015; Wang X. et al., 2016). Most of the QTLs for SL and SPS obtained in this study were consistent with those previously reported. Among them, one QTL for SL (*cqSL.A10-3*) and four for SPS (*cqSPS.A7-3*, *cqSPS.C3-2*, *cqSPS.C3-3*, and *cqSPS.C6-2*) were repeatedly identified in two to three different environments. With the assistance of the *B*. *napus* reference genome “Darmor-*bzh*,” it was possible to compare the QTLs for silique-related traits detected in this study with those detected in previous studies. *cqSL.A10-3*, *cqSPS.A7-3*, *cqSPS.C3-2*, *cqSPS.C3-3*, and *cqSPS.C6-2* overlapped with QTL mapping intervals of Chen et al. (2007), Shi et al. (2009), Luo et al. (2017), and Zhu et al. (2020), respectively. In this study, the 13 consensus QTLs that were stably expressed in different genetic backgrounds and environments were used as the major QTLs for further candidate gene research. Among these 13 consensus QTLs, *cqSB.C7-4*, *cqST.A5-2*, *cqSPS.A7-3*, *cqSPS.C3-3*, *cqSB.C6-1*, and *cqSPS.C6-2* were further integrated into pleiotropic unique QTLs *uqC7-7*, *uqA5-4*, *uqA7-6*, *uqC3-4*, and *uqC6-4* by meta-analysis (Table 4). The genes underlying these consensus QTL might have pleiotropic effects on two to three traits.

Siliques represent a fruit type specific to members of the *Brassicaceae* family that form from the gynoecium after flowering (Seymour et al., 2008). The transformation of the gynoecium into a silique depends on whether the ovule has been successfully fertilized, and this signal may be produced by the pollen grains (Seymour et al., 2013). Successfully fertilized ovules result in developmental alterations of pistils from senescence to growing fruit. Thus, the failure of any part of these successive processes, including gynoecium formation, fertilization and silique growth, that involving cell proliferation, differentiation and expansion, will eventually affect the development of siliques and seeds, which are the determining factors of rapeseed yield. For example, plants with a *BnaC9.SMG7b* deletion exhibit reduced SPS and SL values, which are caused by developmental defects in the formation of functional FGs owing to the interruption of meiotic anaphase II (Li et al., 2015). *BnaC9.SMG7b*, the successfully cloned rapeseed silique gene, also underlies the CI of *cqSL.A10-3* in this study. *LNG1* regulates longitudinal cell elongation in *Arabidopsis*, and *lng1* dominant mutant plants are characterized by elongated siliques owing to longitudinally elongated and transversely narrowed cells in the siliques (Lee et al., 2006). CNGC18 is an essential Ca^2+^ channel for pollen tube guidance in *Arabidopsis*. *CNGC18* point mutations result in shorter siliques, reduced male fertility and fewer seeds per silique (Gao Q. et al., 2016). The *Arabidopsis* gene *STY2* promotes the formation of the apical tissues of the gynoecium. The *sty2* mutant lines exhibit many developmental defects in reproductive tissues, including shortened siliques and aborted ovules (Kuusk et al., 2002). *KIN*βγ, a component of the regulatory subunit of the *SNF1*-related protein kinase, is required for pollen germination on the stigma surface. *KIN*βγ mutants display defects in organogenesis and growth, including shorter statures and siliques (Gao X. et al., 2016). *ABCC13* is expressed in the seed coat and embryo, and its loss of function in *Arabidopsis* leads to decreased silique lengths and seed yields (El Guizani et al., 2014). *ARID1* is required for sperm cell formation in *Arabidopsis*, and the *ARID1* mutant shows reduced seed set and short siliques, which are caused by defects in gametophyte formation owing to an arrested mitotic cell cycle (Zheng et al., 2014). *LAC4*, a laccase gene regulated by miR397b, controls both lignin biosynthesis and seed yield in *Arabidopsis*, and overexpressing miR397b may increase silique lengths and seed sizes by modulating *LAC4* (Wang et al., 2014). *HTH* is involved in regulating floral organ fusion in *Arabidopsis*, and the *hth* mutant shows reduced seed set and short siliques, which are caused by reduced pollen fertility and aborted ovules (Krolikowski et al., 2003). The homozygous *aog1* in *Arabidopsis* shows reduced seed set and short siliques compared with the wild type, and this caused by a significant reduction in fertility owing to reduced pollen formation and severe defects in embryo sacs (Cui et al., 2015). PYL8 is a regulatory component of the ABA receptor, which is vital for regulating seed germination, root and shoot development and abiotic stress responses (Garcia-Maquilon et al., 2021), and a *pyl8* mutant shows an ABA-insensitive phenotype, including a lower seed yield owing to fewer siliques having shorter lengths (Gonzalez-Guzman et al., 2012). *Arabidopsis HMG1* encodes a 3-hydroxy-3-methylglutaryl coenzyme A reductase, which is a key element of the sterol biosynthetic process that is required for cell viability and growth. The T-DNA insertion *hmg1* mutant in *Arabidopsis* shows shorter siliques and fewer seeds per silique compared with the wild type, and this was caused by reduced cell elongation and fertility owing to a reduced sterol level (Suzuki et al., 2004).

## Data Availability Statement

The original contributions presented in the study are included in the article/Supplementary Material, further inquiries can be directed to the corresponding author/s.

## Author Contributions

XZ and KY co-wrote the manuscript. CS, WZ, and FC participated in the field experiment and collected the data. CP, XWu, and RS carried out QTL analysis and prediction of candidate genes. JZ revised the manuscript. XWa designed, led and coordinated the overall study. All authors have read and approved the final manuscript.

## Conflict of Interest

The authors declare that the research was conducted in the absence of any commercial or financial relationships that could be construed as a potential conflict of interest.

## Publisher’s Note

All claims expressed in this article are solely those of the authors and do not necessarily represent those of their affiliated organizations, or those of the publisher, the editors and the reviewers. Any product that may be evaluated in this article, or claim that may be made by its manufacturer, is not guaranteed or endorsed by the publisher.

## Supplementary Material

Supplementary Additional File 1 The 137 identified QTLs and 120 consensus QTLs for five silique-related traits detected in seven environments.

Supplementary Additional File 2 Consensus QTL locations of silique-related traits detected from seven different environments.

Supplementary Additional File 3 The 89 unique QTLs associated with five silique-related traits in the AH population.

Supplementary Additional File 4 Genes underlying the confidence intervals of the 13 stable QTLs.
