# Supplementary figures and images for: QTL Analysis of Five Silique-Related Traits in Brassica napus L. Across Multiple Environments
Source: Front Plant Sci. 2021 Nov 23;12:766271. doi: 10.3389/fpls.2021.766271 (PMC8650614; doi:10.3389/fpls.2021.766271)

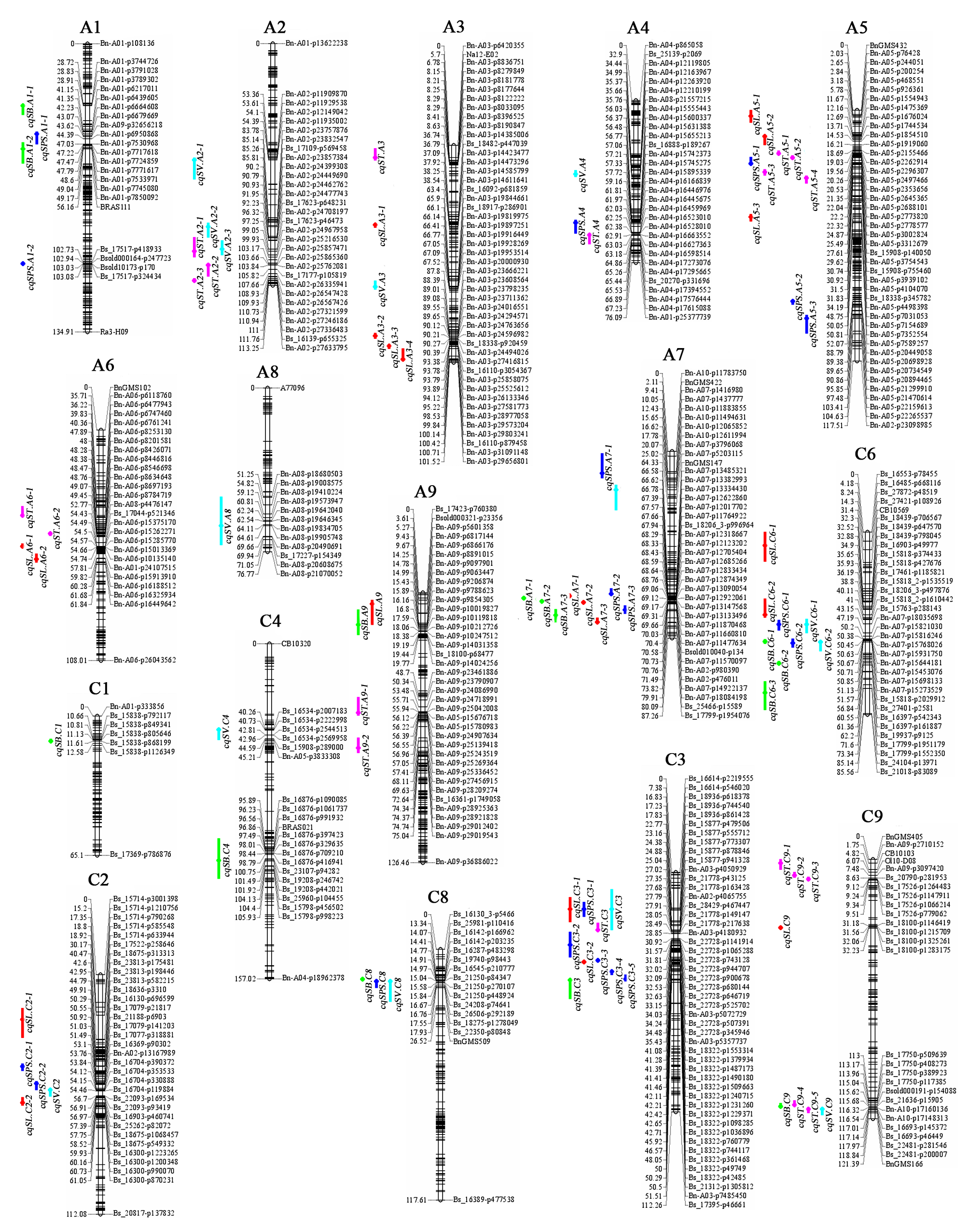

Supplement: Supplementary Additional File 1 — The 137 identified QTLs and 120 consensus QTLs for five silique-related traits detected in seven environments. [file Data_Sheet_1.zip › Additional File 2.tif]
